# L-Serine enables reducing the virulence of *Acinetobacter baumannii* and modulating the SIRT1 pathway to eliminate the pathogen

**DOI:** 10.1128/spectrum.03226-23

**Published:** 2024-01-19

**Authors:** Jianxia Zhou, Dingyun Feng, Xia Li, Yuetao Chen, Min Zhang, Wenbin Wu, Jiaxin Zhu, Hui Li, Xuanxian Peng, Tiantuo Zhang

**Affiliations:** 1 Department of Pulmonary and Critical Care Medicine, The Third Affiliated Hospital of Sun Yat-Sen University, Institute of Respiratory Disease of Sun Yat-Sen University, Guangzhou, People's Republic of China; 2 School of Life Sciences, Sun Yat-Sen University, Guangzhou, People's Republic of China; Universidad Andres Bello, Santiago, Chile

**Keywords:** GC-MS, *Acinetobacter baumannii*, *G. mellonella*, virulence, SIRT1

## Abstract

The emergence of high-virulent *Acinetobacter baumannii* strains increases the mortality of patients and seriously affects their prognosis, which motivates us to explore novel ways to control such infections. In this study, gas chromatography–mass spectrometry was adopted to explore the metabolic difference between high- and low-virulent *A. baumannii* strains, and the decreased L-serine levels were identified as the most crucial biomarker in low-virulent *A. baumannii* strains. *In vitro*, L-serine reduced the virulence of *A. baumannii* to Beas 2B cells and inhibited the activation of NLRP3 inflammasome via decreasing the generation of ROS and mtROS and the release of inflammatory cytokines (IL-18 and IL-1β) through upregulating SIRT1. *In vivo*, the *Galleria mellonella* model was adopted. L-serine downregulated the levels of virulence genes (*ompA*, *carO*, and *omp*33-36), reduced the mortality of *A. baumannii* to *G. mellonella*, and decreased the blacking speed as well as the degree of *G. mellonella* after infection. Taken together, we found that L-serine can reduce the virulence of *A. baumannii* and enhance the host’s defense against the pathogen, providing a novel strategy for the treatment of infections caused by *A. baumannii*.

**IMPORTANCE:**

*Acinetobacter baumannii* has become one of the most common and severe opportunistic pathogens in hospitals. The high-virulent *A. baumannii* strains pose a great threat to patients and increase the risk of nosocomial infection. However, the mechanism of virulence in *A. baumannii* is still not well understood. In the present study, we identified potential biomarkers in low-virulent *A. baumannii* strains. Our analysis revealed the effect of L-serine on reducing the virulence of *A.baumannii*. This discovery suggests that targeting L-serine could be a promising strategy for the treatment or adjunctive treatment of *A. baumannii* infections. The development of treatments targeting virulence may provide a substitute for the increasingly failed traditional antibacterial treatment.

## INTRODUCTION

*Acinetobacter baumannii* is an oxidase-negative and Gram-negative bacterium, which has become one of the most common opportunistic pathogens in hospitals, especially in intensive care units (1). *A. baumannii* exists widely in various environments, including the surfaces of medical facilities, posing a great threat to nosocomial infections. The emergence of high-virulent *A. baumannii* strains poses a serious threat to patients and contributes to the increased ocurrence of complications, such as ventilator-associated pneumonia (VAP). VAP not only prolongs hospital stay but also imposes a high financial burden and increases the risk of death (2). Therefore, it is clinically urgent to explore new approaches to control the virulence of *A. baumannii*.

Besides antibiotic-based elimination, approaches to reducing bacterial virulence are especially recommended to avoid the development of antibiotic resistance. Moreover, the approaches not only make pathogens less virulent directly but also enhance the host’s ability to fight infections. A recently developed reprogramming metabolomic approach provides a solid and feasible way to solve the question. The reprogramming metabolomics identify biomarkers as reprogramming metabolites through comparison between abnormal (for example, serum resistance and antibiotic resistance) metabolome and control metabolome. Then, the reprogramming metabolites were used to implement a reversal of the abnormal metabolic state into the normal or normal-similar metabolic state, thereby leading to changes in biological phenotypes such as reversal of serum resistance and antibiotic resistance (3, 4). Using this approach, we have demonstrated that serum resistance, a pathogenic feature, is modulated by glycine, serine, and threonine metabolism. Exogenous glycine restores the depression of glycine, serine, and threonine metabolism in serum-resistant bacteria, enabling the killing of the serum-resistant bacteria by complement (5). Similarly, exogenous glutamine promotes β-lactam-, aminoglycoside-, quinolone-, and tetracycline-induced killing of *Escherichia coli* and potentiates ampicillin to eliminate multidrug-resistant *Pseudomonas aeruginosa, Acinetobacter baumannii, Klebsiella pneumoniae, Edwardsiella tarda, Vibrio alginolyticus,* and *Vibrio parahaemolyticus*. On the other hand, reprogramming metabolites have been found to restore the host’s ability to eliminate bacterial pathogens (6). However, most studies on reprogramming metabolomics have focused either on the bacterial perspective or the host perspective, and there is a lack of simultaneous studies examining both effects. Furthermore, information regarding reprogramming metabolomics-mediated virulence reduction is also limited.

Here, the reprogramming metabolomic approach was used to explore the metabolic characteristics between high- and low-virulent *A. baumannii* strains. This led to the identification of L-serine as the most key biomarker. Exogenous L-serine reduced the virulence of these high- and low-virulent *A. baumannii* strains. Furthermore, taking the L-serine-activated expression of SIRT1 in the C2C12 myotube as a clue (7), the effect of exogenous L-serine on the elimination of *A. baumannii* strains was investigated in Beas 2B cells. It was shown that L-serine inhibited the activation of the NLRP3 inflammasome and decreased inflammatory cytokines through upregulating SIRT1, which is responsible for the elimination.

## RESULTS

### Virulence of *A. baumannii* strains

**Fig 1:**
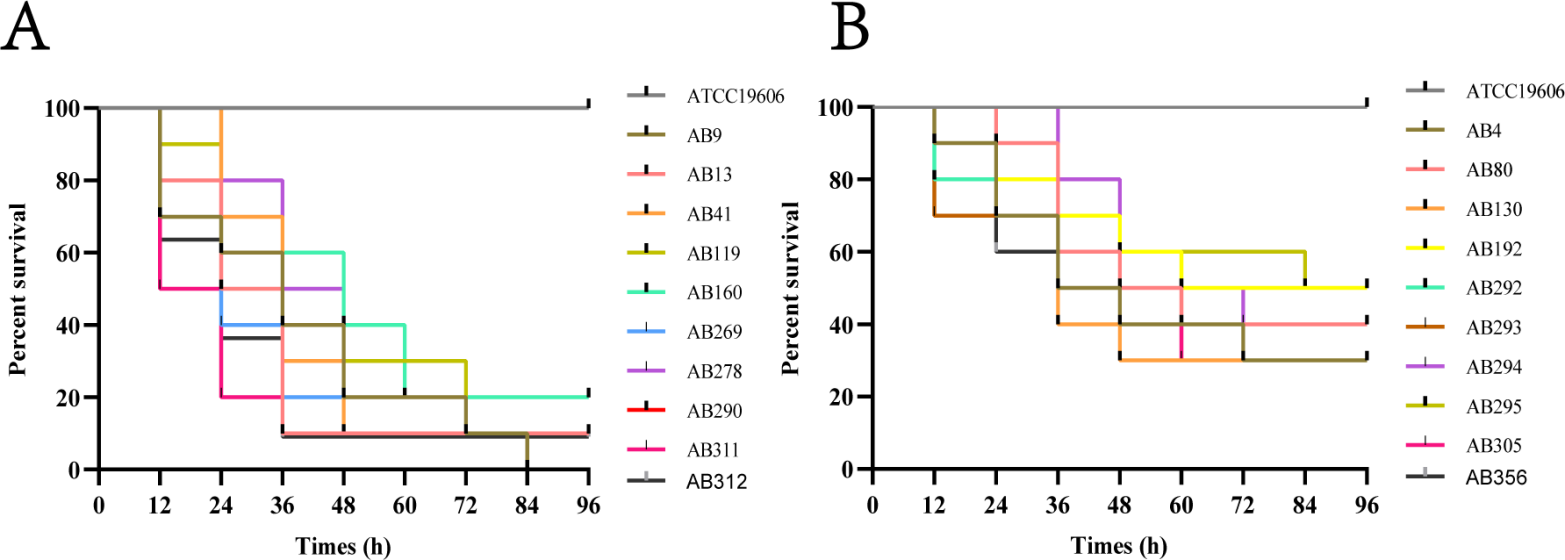
Assessing the virulence of *A. baumannii* strains using the *G. mellonella* model. (**A**) Survival rates of *G. mellonella* infected with high-virulent *A. baumannii* strains. (**B**) Survival rates of *G. mellonella* infected with low-virulent *A. baumannii* strains.

To assess the virulence of the 20 *A. baumannii* strains included in this study, the *Galleria mellonella* virulence assay was adopted. The results revealed varying levels of virulence toward *G. mellonella*. The information on these selected strains is summarized in Table 1. Specifically, 10 strains (AB9, AB13, AB41, AB119, AB160, AB269, AB278, AB290, AB311, and AB312) exhibited a mortality rate of ≥80%, indicating high virulence (Fig. 1A). On the other hand, the remaining strains (AB4, AB80, AB130, AB192, AB292, AB293, AB294, AB295, AB305, and AB356) displayed a mortality rate of <80%, indicating low virulence (Fig. 1B).

**TABLE 1:** The virulence of *A. baumannii* strains^*a*^

Strain no.	Meropenem	Piperacillin–tazobactam	Tigecycline	Mortality	Virulence
AB 4	R	R	S	70%	L
AB 9	R	R	S	100%	H
AB 13	R	R	S	90%	H
AB 41	R	R	S	90%	H
AB 80	S	S	S	60%	L
AB 119	R	R	S	90%	H
AB 130	S	S	S	70%	L
AB 160	S	S	S	80%	H
AB 192	R	R	S	50%	L
AB 269	R	R	S	90%	H
AB 278	R	I	S	80%	H
AB 290	S	S	S	90%	H
AB 292	R	R	I	60%	L
AB 293	R	R	S	50%	L
AB 294	R	R	S	60%	L
AB 295	R	R	S	50%	L
AB 305	R	R	S	70%	L
AB 311	S	S	S	90%	H
AB 312	R	R	S	90%	H
AB 356	R	R	R	70%	L

^*a*^ H: high-virulent; L: low-virulent; R: resistant; S: sensitive; I: intermediate.

### Metabolic profiles in low- and high-virulent *A. baumannii* strains

**Fig 2:**
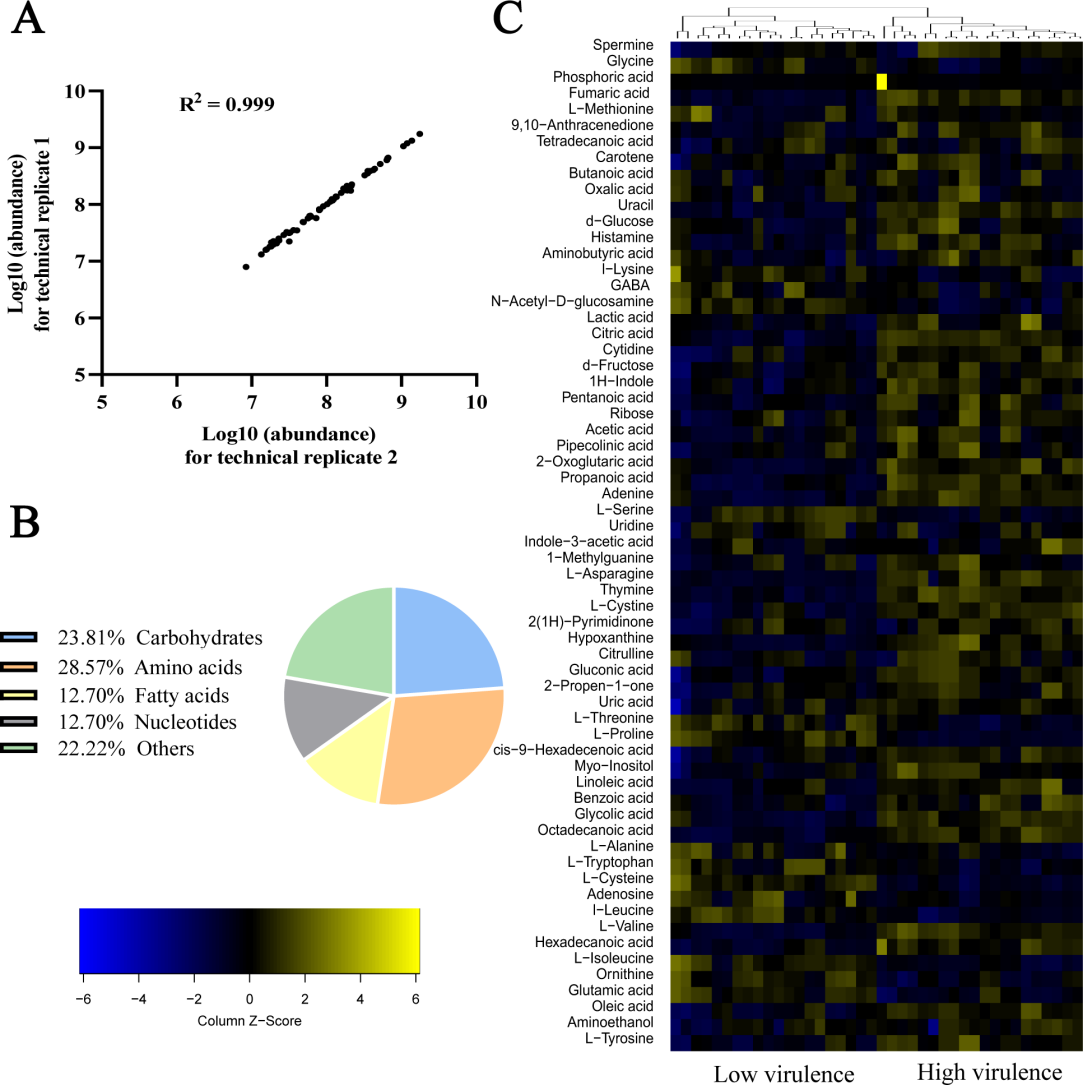
Metabolic profile of high- and low-virulent *A. baumannii* strains. (**A**) Pearson correlation coefficient of technical replicates. (**B**) Categories of all identified metabolites. (**C**) Heatmap of unsupervised hierarchical clustering of all metabolites. Yellow and blue colors indicate increased and decreased levels of the metabolites, respectively.

To understand the metabolic profiles between low- and high-virulent *A. baumannii* strains, a gas chromatography–mass spectrometry (GC-MS)-based metabolomic approach was employed. Each group was carried out with 10 biological samples as well as two technical replicates, resulting in a total of 40 data sets. The correlation coefficients between the technical replicates ranged from 0.93 to 0.99, indicating the repeatability and reliability of the data (Fig. 2A). A total of 63 metabolites were identified in each group after removing interference and internal standard ribitol peaks. These metabolites were categorized into carbohydrate (23.81%), amino acid (28.57%), nucleotide (12.70%), fatty acid (12.70%), and other metabolites (22.22%) (Fig. 2B). High- and low-virulent strains were clustered separately and represented as a heatmap (Fig. 2C). The heatmap clearly shows that low-virulent strains are predominantly depicted in blue, indicating a lower metabolic state, and high-virulent strains are predominantly depicted in yellow, indicating a higher metabolic state. These findings suggest that high-virulent and low-virulent strains exhibit distinct metabolic profiles.

### Differential metabolomes between low- and high-virulent *A. baumannii* strains

**Fig 3:**
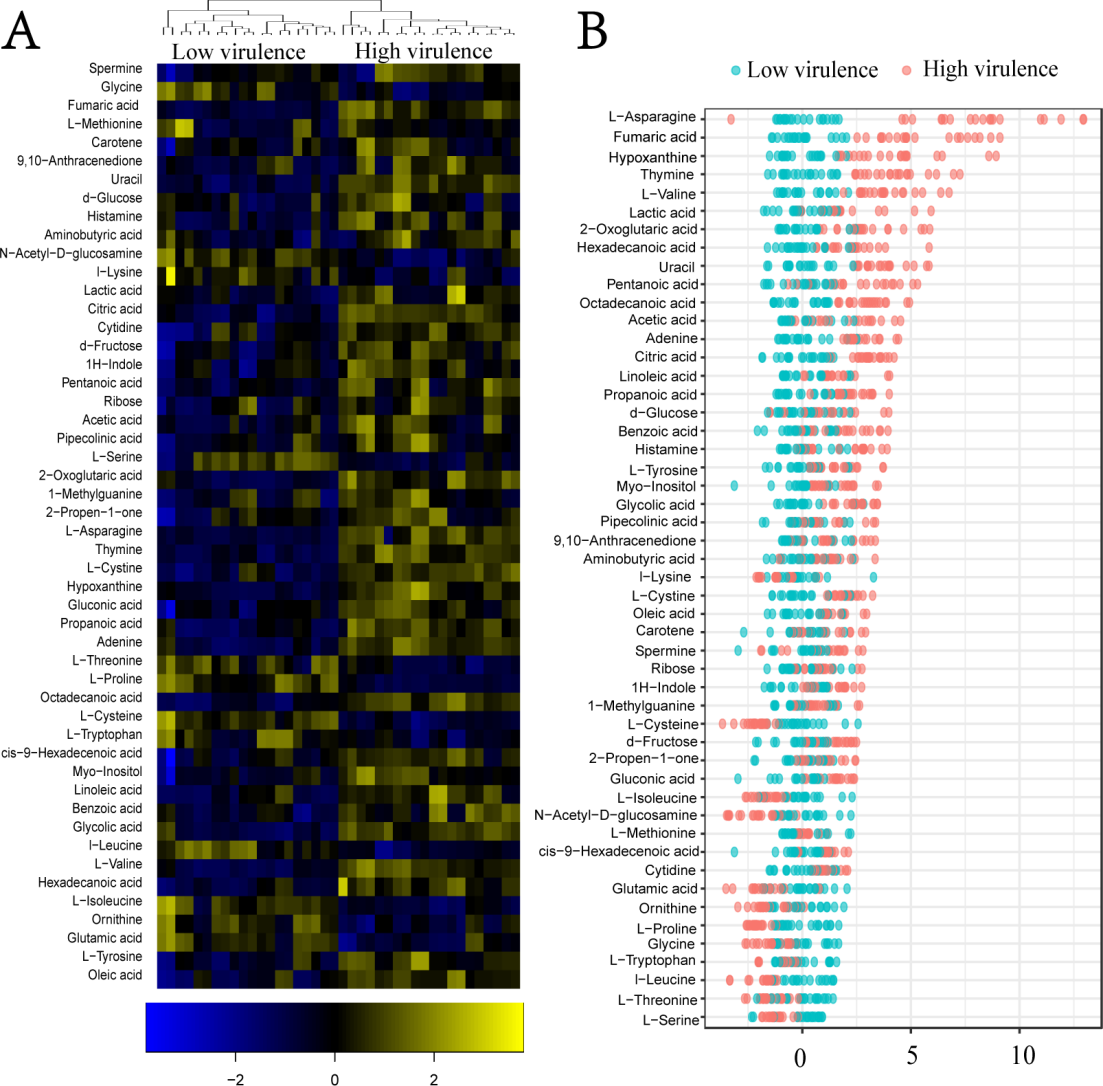
Differential metabolomic analysis of high- and low-virulent *A. baumannii* strains. (**A**) Heatmap illustrating the differential metabolites. Blue and yellow colors indicate decreased and increased metabolite levels, respectively. (**B**) *Z*-score plot displaying the changing levels of differential metabolites. Each dot represents a metabolite in one technical replicate.

When the Kruskal–Wallis test was adopted to explore differential metabolites between two groups (*P* < 0.05), 50 differential metabolites were identified out of 63 metabolites (Fig. 3A). The low-virulent strains show lower metabolic states than high-virulent strains mostly. A *Z*-score plot was used to clearly demonstrate the varying levels of metabolites between the two groups (Fig. 3B). The *Z*-values ranged from −3.85 to 6.06. In the high-virulent group, the top five increased metabolites were L-asparagine, fumaric acid, hypoxanthine, thymine, and L-valine, while the top five decreased metabolites were L-serine, L-threonine, L-leucine, L-typtophan, and glycine. Among the decreased metabolites, L-serine, L-threonine, and L-glycine work for glycine, serine, and threonine metabolism. These findings not only suggest that high-virulent strains have higher metabolic states but also reveal that the levels of L-serine, L-threonine, and L-glycine are low in high-virulent strains.

### Enriched pathways in low- and high-virulent *A. baumannii* strains

**Fig 4:**
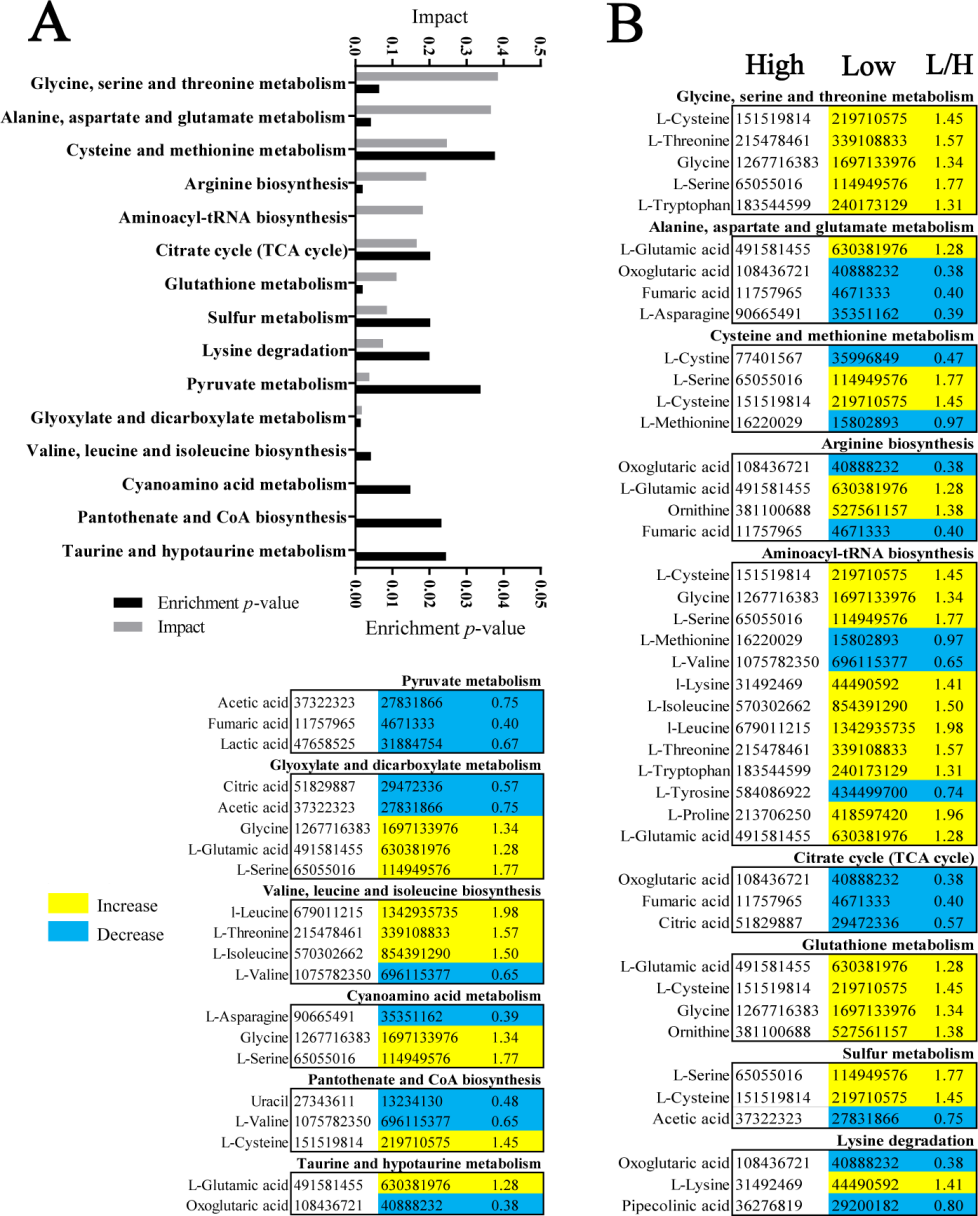
Enriched pathways in high- and low-virulent *A. baumannii* strains. (**A**) Pathway enrichment analysis of differential metabolites between high- and low-virulent *A. baumannii* strains (*P* < 0.05). (**B**) Integrative analysis of differential metabolites in significant pathways (*P* < 0.05). The terms “low” and “high” refer to low-virulent and high-virulent strains, respectively. Increased and decreased metabolites are represented by yellow and blue colors, respectively.

Pathway enrichment plays a crucial role in exploring changes in metabolic pathways, and in our current study, it has led to the identification of 15 enriched pathways (Fig. 4A). Based on their impact, glycine, serine, and threonine metabolism; alanine, aspartate, and glutamate metabolism; and cysteine and methionine metabolism were the top three enriched metabolic pathways. All of these pathways are related to amino acid metabolism. The metabolites involved in each enriched pathway are listed in Fig. 4B. All metabolites in the glycine, serine, and threonine metabolism pathway were higher in low-virulent than high-virulent *A. baumannii* strains, while most metabolites were lower in the aspartate and glutamate metabolism pathway, and half of the metabolites were either higher or lower in the cysteine and methionine metabolism pathway (Fig. 4B). These findings highlight the significance of glycine, serine, and threonine metabolism in distinguishing between high-virulent and low-virulent strains.

### Identification of biomarkers differentiating high-virulent and low-virulent strains

**Fig 5:**
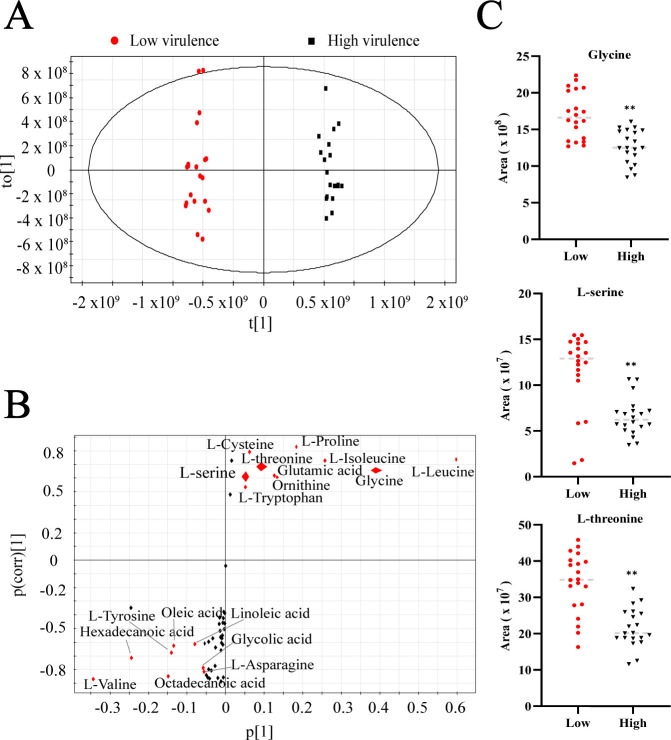
Biomarkers for the virulence of *A. baumannii* strains. (**A**) OPLS-DA of metabolomes in high-virulent and low-virulent *A. baumannii* strains. The black and red dots represent the biological and technical replicate analysis of high- and low-virulent strains, respectively. (**B**) S-plot generated by OPLS-DA. The predictive indicator *P*[1] and correlation *P*(corr)[1] were used to distinguish low-virulent strains. Metabolites with absolute *P* and absolute *P*(corr) values greater than or equal to 0.05 and 0.5, respectively, were considered potential biomarkers and are marked in red. (**C**) Scatter plot of metabolites in the glycine, serine, and threonine metabolism pathways. ^**^P < 0.01.

Orthogonal partial least-squares discriminant analysis (OPLS-DA) was employed to investigate the sample patterns of the two metabolomes (Fig. 5A). High-virulent and low-virulent strains were separated by *t*[1]. The red dots represent low-virulent clusters, and the black dots represent high-virulent clusters. Additionally, the S-plot was adopted to identify discriminating variables (Fig. 5B). In this plot, both *P*[1] (with an absolute value no less than 0.05) and *P*(corr)[1] (with an absolute value no less than 0.5) were considered important indicators for screening the biomarkers that distinguish high-virulent from low-virulent strains. Biomarkers with higher relevance and larger weights were marked by red. Eight downregulated metabolites (L-valine, octadecanoic acid, L-asparagine, glycolic acid, linoleic acid, oleic acid, L-tyrosine, and hexadecanoic acid) and ten upregulated metabolites (L-serine, L-cysteine, L-threonine, L-tryptophan, glutamic acid, ornithine, L-proline, L-isoleucine, glycine, and L-leucine) were identified as biomarkers in low-virulent compared with high-virulent *A. baumannii* strains. Among these biomarkers, glycine, serine, and threonine were included. The abundance levels of glycine, serine, and threonine were displayed by scatter plots in Fig. 5C. Since serine was significantly decreased in high-virulent strains, suggesting that depression is needed for high virulence, decreased serine was selected as a crucial biomarker for reducing high virulence.

### L-Serine reduces the expression of virulence genes of *A. baumannii*

**Fig 6:**
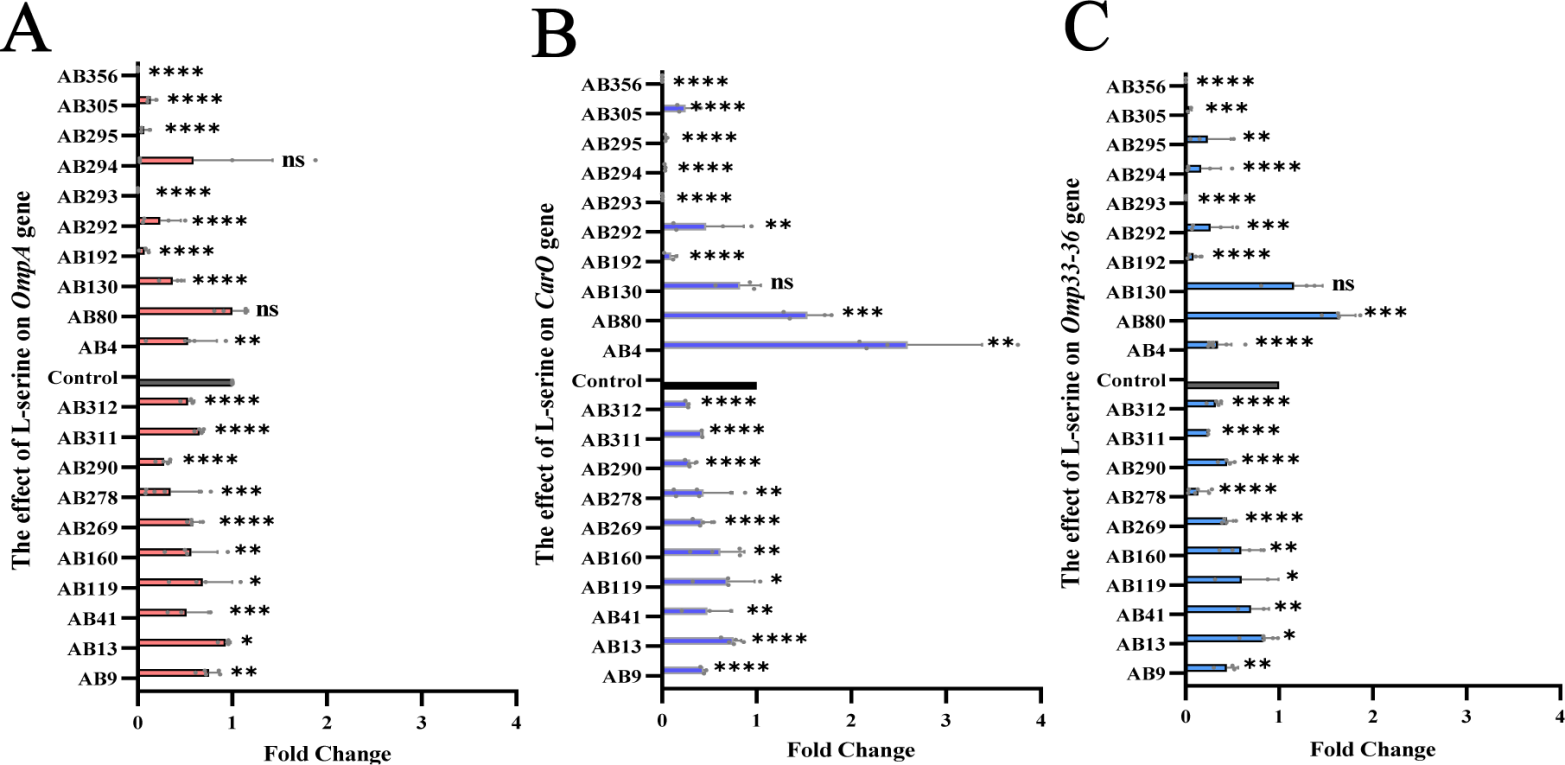
Decreased virulence gene levels of *A. baumannii* strains by L-serine. (**A**) Effect of L-serine on the expression levels of *OmpA* gene. (**B**) Effect of L-serine on the expression levels of *Caro* gene. (**C**) Effect of L-serine on the expression levels of *Omp*33-36 gene. “ns” indicates no significant difference. ^*^P < 0.05. ^**^P < 0.01. ^***^P < 0.001. ^****^P < 0.0001.

To explore the impact of L-serine on reducing the virulence of *A. baumannii* strains, virulence genes *ompA*, *carO*, and *omp*33-36 were selected. In order to do this, *A. baumannii* strains were cultured with or without L-serine (4 mg/mL) for 24 hours. The cells were then collected for quantitative reverse transcription–PCR (qRT-PCR) analysis to measure the expression levels of *ompA*, *carO*, and *omp*33-36. The results showed that the addition of exogenous L-serine significantly downregulated the expressions of *ompA*, *carO*, and *omp*33-36 in all high-virulent *A. baumannii* strains (*P*＜0.05). Furthermore, the addition of L-serine also downregulated *ompA*, *carO*, and *omp*33-36 by 80%, 70%, and 80%, respectively, in low-virulent *A. baumannii* strains (Fig. 6A through C). These findings demonstrate that L-serine effectively reduces the expression of *ompA*, *carO*, and *omp*33-36.

#### L-Serine reduces the virulence of *A. baumannii*

**Fig 7:**
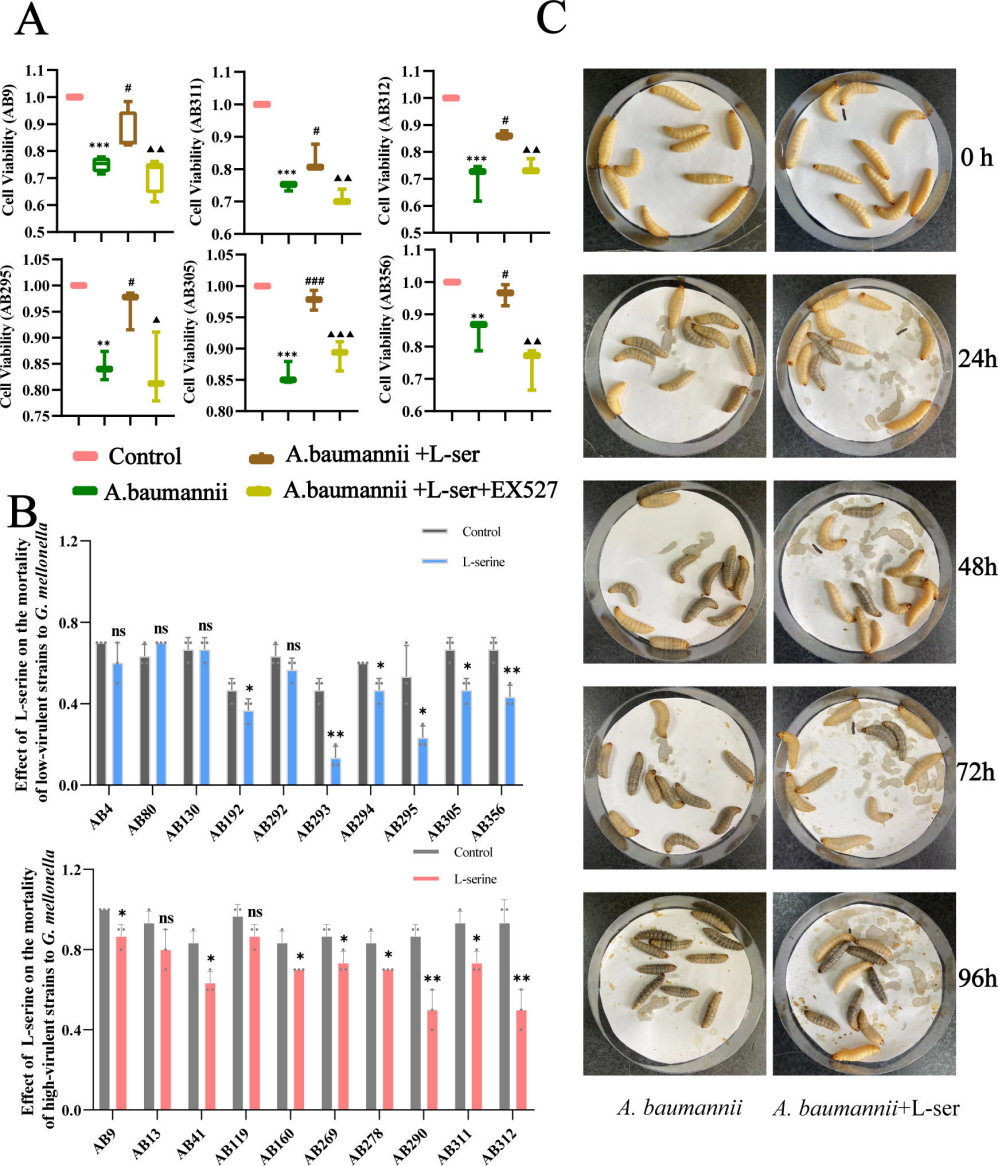
L-Serine reduces the virulence of *A. baumannii*. (**A**) L-Serine attenuates the cell damage induced by *A. baumannii* (AB9, AB311, AB312, AB295, AB305, and AB356). (**B**) L-Serine reduced the virulence of high- and low-virulent *A. baumannii* strains. (**C**) The effect of L-serine on the mortality of *G. mellonella* using the AB13 strain as an example. “ns” indicates no significant difference. ^*, #^*P* < 0.05. ^**,▲▲^*P* < 0.01. ^***^P < 0.001. ^*^Compared with the control group. ^#^Compared with the *A. baumannii* group. ^▲^Compared with the *A. baumannii*+L-serine group.

The CCK-8 test was used to determine whether L-serine can repair the damage caused by *A. baumannii* to Beas 2B cells. In this experiment, three strains of *A. baumannii* with high virulence (AB9, AB311, and AB312) and three with low virulence (AB295, AB305, and AB356) were randomly selected for verification. Beas 2B cells were infected with *A. baumannii* and treated with or without L-serine. It was found that *A. baumannii* reduced cell viability compared to the control group. However, the addition of L-serine elevated cell viability in almost all strains selected (*P*＜0.05) (Fig. 7A). Reports have indicated that L-serine regulates SIRT1, which mediates anti-inflammation (7–9). This motivated us to test whether the repair mediated by L-serine is related to the activation of SIRT1. As anticipated, L-serine did not improve the cell viability due to pretreatment with EX527, an inhibitor of SIRT1 (*P*＜0.05). These data indicate that L-serine decreases the virulence of *A. baumannii* to Beas 2B cells through upregulating SIRT1 (Fig. 7A).

Furthermore, the *G. mellonella* model was used to evaluate the effect of L-serine on the virulence of *A. baumannii*. First, an appropriate concentration of L-serine that did not damage the activity of *G. mellonella* was tested, which led to 4 mg/mL L-serine as a suitable concentration (Fig. S1). Exogenous L-serine decreased the mortality of *G. mellonella* in 60% low-virulent strains (AB192, AB293, AB294, AB295, AB305, and AB356) and 80% high-virulent strains (AB9, AB41, AB160, AB269, AB278, AB290, AB311, and AB312) (*P* < 0.05) (Fig. 7B). Moreover, L-serine limited the blackening speed and corruption degree of *G. mellonella* to varying degrees compared to the single *A. baumannii* infection group (AB4, AB80, AB130, AB292, AB13, and AB119) (Fig. 7C). These results indicate that L-serine reduces the virulence of *A. baumannii* to both Beas 2B cells and *G. mellonella*.

### L-Serine restores SIRT1 level inhibited by *A. baumannii*

**Fig 8:**
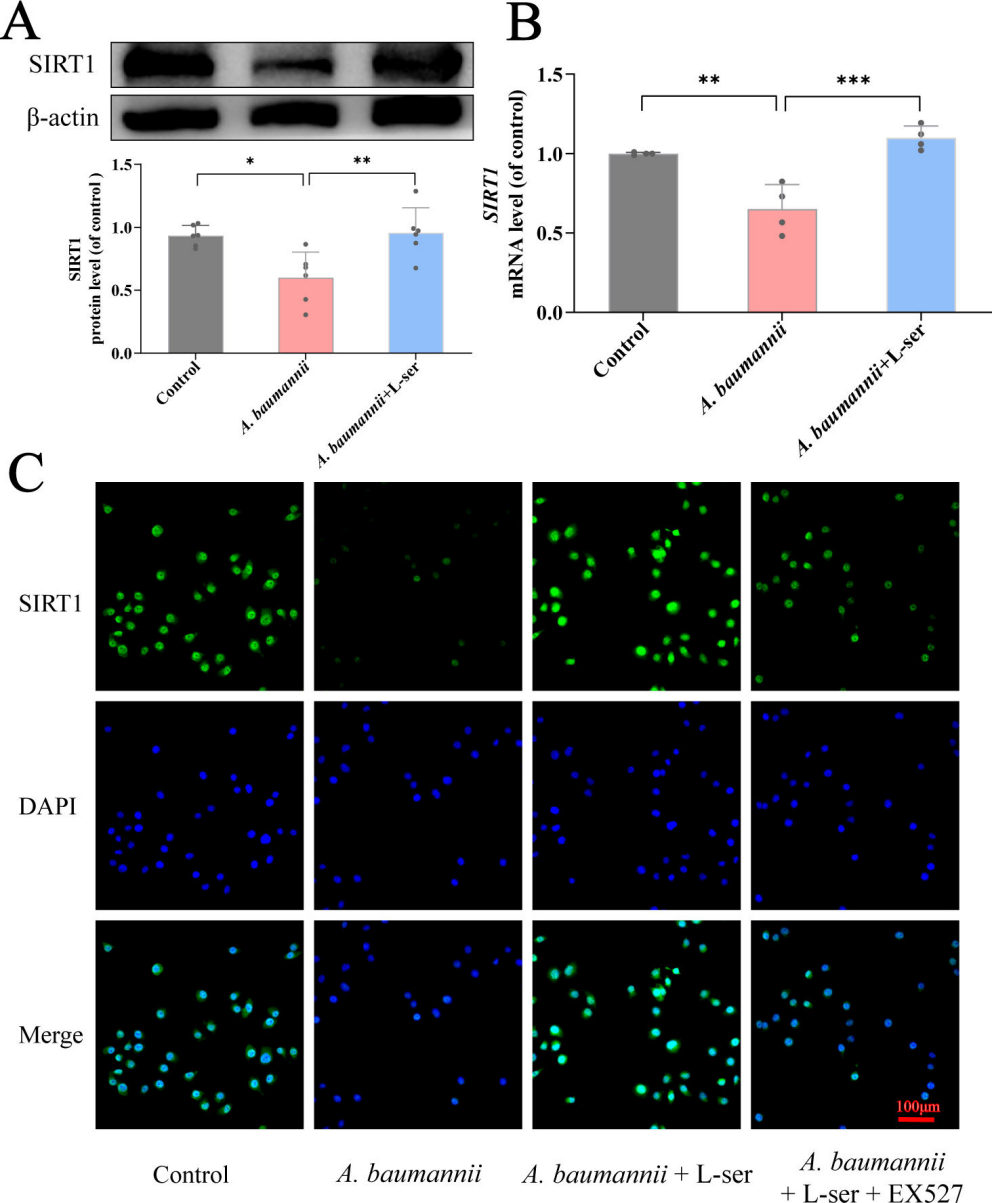
Expression levels of SIRT1 in Beas 2B cells. (**A**) Beas 2B cells were treated with *A. baumannii* and L-serine. The expression of SIRT1 was detected by immunoblotting, with β-actin used as a loading control. It was quantified and normalized to the β-actin values. (**B**) Beas 2B cells were treated as described above. The expression of SIRT1 was measured by qRT-PCR. It was quantified and normalized to the GAPDH values. (**C**) Immunofluorescence staining was performed to observe the expression of SIRT1 (green) in Beas 2B cells, with the nuclei stained with DAPI (blue). Scale bar, 100 μm. Data are presented as the mean ± SD. ^*^P < 0.05. ^**^P < 0.01. ^***^P < 0.001. ^*^Compared with the control group.

Following the clue that L-serine-mediated repair is related to the activation of SIRT1, we investigated the changes of SIRT1 at the protein and mRNA levels in the presence of exogenous L-serine. Beas 2B cells were treated with 2, 4, or 6 mg/mL L-serine, and the abundance of SIRT1 was measured by western blot (Fig. S2A). The abundance was elevated in an L-serine dose-dependent manner from 4 mg/mL. Similarly, qRT-PCR analysis showed that the expression of genes encoding SIRT1 increased with an increasing dose of L-serine from 4 mg/mL (Fig. S2B). Therefore, 4 mg/mL of L-serine was selected to examine whether exogenous L-serine could restore the reduced abundance and gene expression level of SIRT1 inhibited by *A. baumannii*. Beas 2B cells infected with *A. baumannii* showed a decrease in SIRT1 abundance and gene expression level. However, these cells restored the abundance and expression with the addition of L-serine (Fig. 8A and B). Furthermore, the immunofluorescence assay was conducted to observe the subcellular location and level of SIRT1 in Beas 2B cells. Luciferase activity was reduced and restored in the absence or presence of L-serine, respectively, when Beas 2B cells were infected with *A. baumannii*. However, the restoration was inhibited by EX527 (Fig. 8C). These results suggest that SIRT1 in the nucleus is decreased due to *A. baumannii* infection, which can be recovered by the addition of L-serine.

### L-Serine removes ROS/mtROS and mitochondrial damage caused by *A. baumannii*

**Fig 9:**
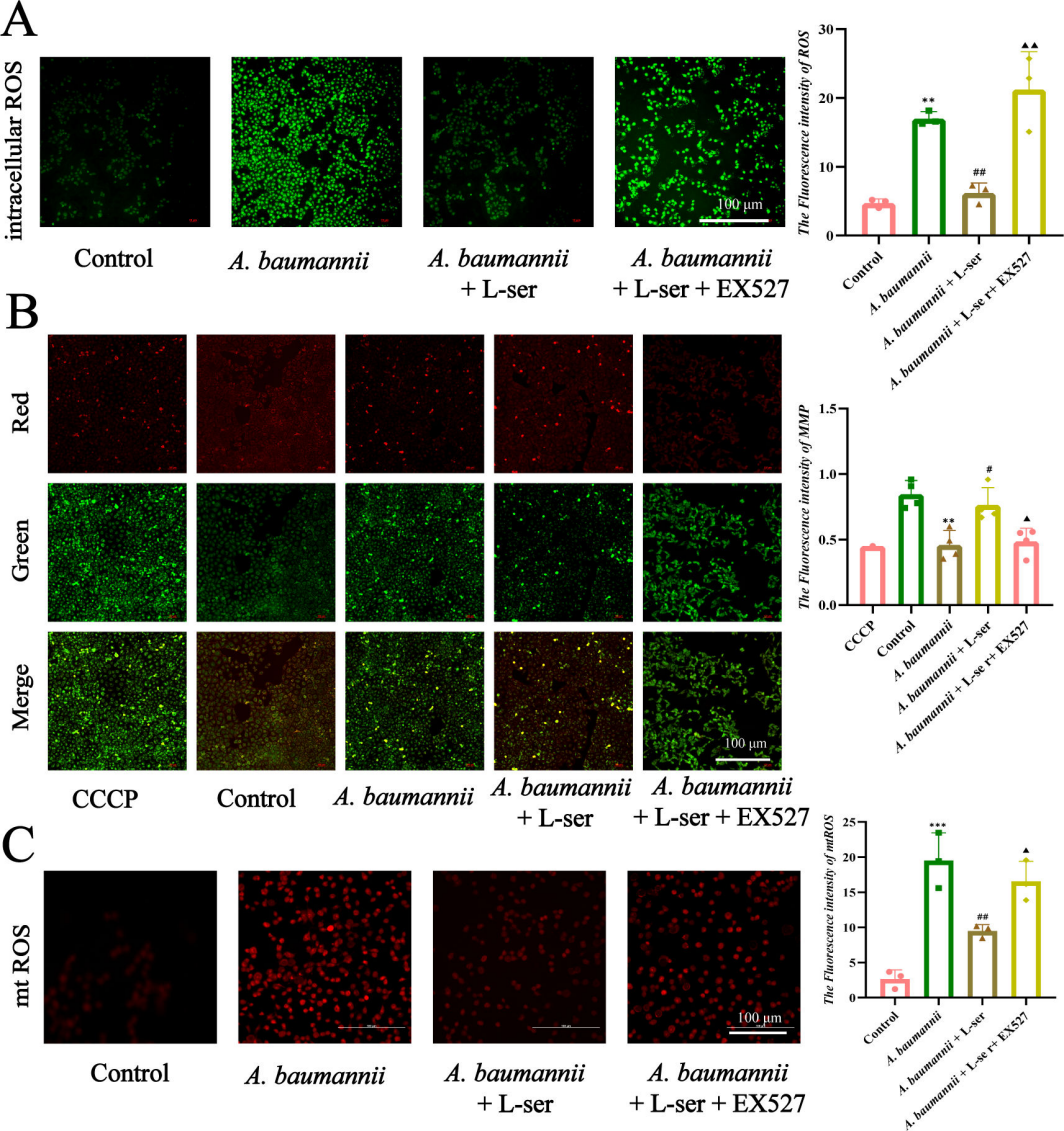
L-Serine inhibits intracellular ROS and mtROS levels and repairs MMP by regulating SIRT1 in Beas 2B cells. (**A**) Intracellular ROS levels were detected using DCFH-DA probe in different groups, and the fluorescence intensity was quantified using ImageJ. (**B**) Changes in MMP were assessed using the JC-1 probe, and the ratio of red to green fluorescence was analyzed. (**C**) mtROS levels in Beas 2B cells were detected and quantified in different groups. Scale bar, 100 µm. ^#,▲^*P* < 0.05. ^**, ##,▲▲^*P* < 0.01. ^***^P < 0.001. ^*^Compared with the control group. ^#^Compared with the *A. baumannii* group. ^▲^Compared with the *A. baumannii*+L-serine group.

Reports have indicated that SIRT1 plays a role in anti-inflammation by reducing ROS levels, which can cause mitochondrial dysfunction. In order to investigate whether L-serine-induced reduction in *A. baumannii* is related to the ROS mechanism in Beas 2B cells, the cells were infected with *A. baumannii*. ROS, mtROS, and mitochondrial dysfunction were detected in the absence or presence of L-serine or/and EX527. The results showed that the increase of ROS induced by *A. baumannii* infection was reversed by L-serine (Fig. 9A). We also examined the effect of L-serine on mitochondrial dysfunction caused by *A. baumannii* by measuring mitochondrial membrane potential (MMP) and mtROS in Beas 2B cells. For the measurement of MMP, CCCP was used as a control, which causes MMP loss and results in the formation of JC-1 monomer, indicating mitochondrial damage. The addition of CCCP was similar to the infection caused by *A. baumannii*, showing green fluorescence. On the contrary, exogenous L-serine led to red fluorescence, similar to the normal control. Interestingly, the red fluorescence was reversed into green fluorescence when EX527 was added (Fig. 9B). For the measurement of mtROS, which is determined by fluorescence intensity, the strongest and weakest fluorescence was detected in the presence of *A. baumannii* and then plus L-serine, respectively, compared with the control, while middle fluorescence was measured in the presence of *A. baumannii* with L-serine plus EX527 (Fig. 9C). These results indicate that L-serine upregulates SIRT1 to remove ROS/mtROS and mitigate mitochondrial damage caused by *A. baumannii* in Beas 2B cells.

### L-Serine inhibits the inflammation levels caused by *A. baumannii* through upregulating SIRT1

**Fig 10:**
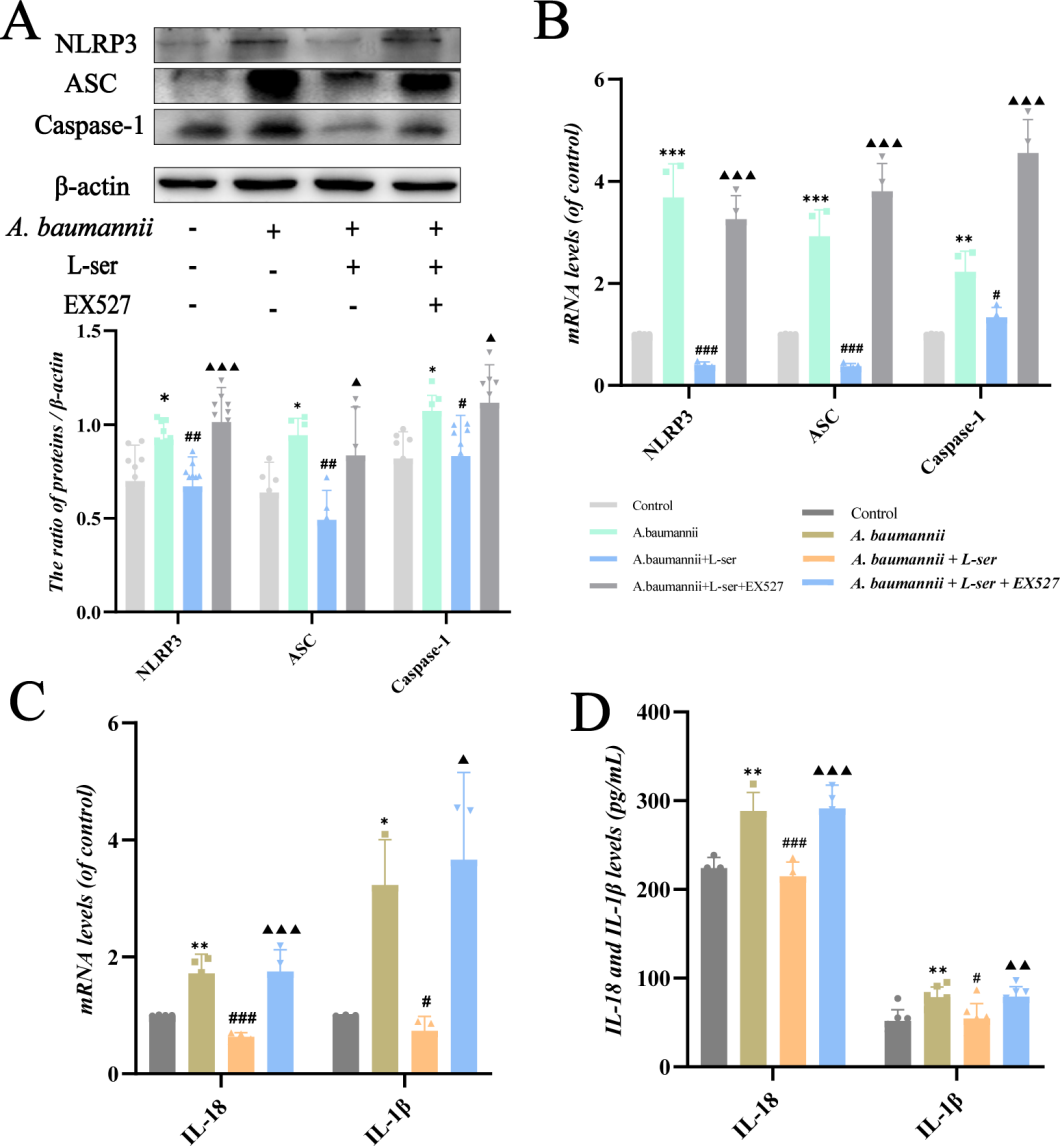
L-Serine was found to inhibit the inflammation levels caused by *A. baumannii* by upregulating SIRT1 in Beas 2B cells. (**A and B**) L-Serine regulates NLRP3, ASC, and caspase-1 at both protein and mRNA levels. (**C**) The effect of L-serine on mRNA levels of IL-18 and IL-1β induced by *A. baumannii*. (**D**) The effect of L-serine on the production of IL-18 and IL-1β caused by *A. baumannii* was measured by ELISA. ^*,#,▲^*P* < 0.05. ^**,##,▲▲^*P* < 0.01. ^***,###,▲▲▲^*P* < 0.001. ^*^Compared with the control group. ^#^Compared with the *A. baumannii* group. ^▲^Compared with the *A. baumannii*+L-serine group.

To further explore the effect of L-serine on the virulence of *A. baumannii*, we examined the inflammation levels in Beas 2B cells. Immunoblot analysis and qRT-PCR results showed that the levels of NLRP3, ASC, and caspase-1 were significantly higher in the *A. baumannii* group but were downregulated by the intervention of L-serine. However, when the combination of L-serine and EX527 was used at the same time, these inflammatory markers were increased again (Fig. 10A and B). We also examined the changes in downstream inflammatory factors at the mRNA levels. The production of IL-18 and IL-1β was stimulated by *A. baumannii* but decreased by L-serine (Fig. 10C). Similar results were observed by the enzyme-linked immunosorbent assay (ELISA). When EX527 was used, the anti-inflammatory effect of L-serine was offset (Fig. 10D). These findings demonstrate that L-serine inhibits the activation of NLRP3-related pathways induced by *A. baumannii* and reduces the inflammation levels through upregulating SIRT1.

## DISCUSSION

With the increase of high-virulent and drug-resistant *A. baumannii* strains, it is of great significance to explore new approaches for preventing and controlling *A. baumannii* infections. In recent years, non-antibiotic therapy has gradually become the focus of research attention, particularly reprogramming metabolomics, which identifies non-toxic nutrient metabolites to reverse bacterial pathogenesis and, in turn, eliminate pathogens (6, 10). We have utilized this approach to demonstrate a link between bacterial antibiotic tolerance/serum resistance and metabolic state and have identified glucose and glycine as effective metabolites for reversing tolerance/serum resistance (5, 11, 12). However, whether the approach reduces bacterial toxicity is not reported. Therefore, in this study, the metabolic states/metabolomes were compared between clinically isolated *A. baumannii* with high and low virulence, and serine was identified as the most crucial biomarker. Exogenous serine not only reduces the virulence of *A. baumannii* to both Beas 2B cells and *G. mellonella* but also alleviates the toxicity caused by *A. baumannii* by upregulating SIRT1, which is negatively regulated by the pathogen. These findings not only provide a non-antibiotic therapy for eliminating refractory *A. baumannii* but also expand the scope of reprogramming metabolic state/metabolomics by reducing bacterial virulence and alleviating toxicity.

Our team has been devoted to analyzing the metabolomics of bacteria and investigating the mechanisms by which exogenous metabolites restore antibiotic sensitivity and kill bacteria (3, 4). In this study, we examined the metabonomic differences between *A. baumannii* with different virulence based on metabonomics and found that the glycine, serine, and threonine metabolism pathway was obviously enriched. Through OPLS-DA, we found that a high abundance of L-serine can serve as a biomarker for identifying low-virulent from high-virulent *A. baumannii* strains, which provides an important clue for our subsequent research. L-Serine is catabolized to pyruvate via gluconeogenesis and converted to lactate through the TCA cycle, during which NADH is oxidized to NAD^+^ (7). NAD^+^ plays a vital role in anti-inflammatory defense systems and antioxidant processes (13). Evidence has shown that SIRT1 is an NAD^+^-dependent deacetylase and regulates the inflammatory process in endothelial cells (14). Here, we prove that L-serine increases the expression level of SIRT1 in Beas 2B cells and plays an important role in reducing the virulence of *A. baumannii*.

*In vitro*, we observed that *A. baumannii* caused a series of infections and injuries in Beas 2B cells due to the downregulation of SIRT1, which can be reversed by L-serine. On the one hand, the activation of NLRP3 inflammasome is crucial in defending against pathogenic infections (15). However, the excessive activation of the NLRP3 inflammasome can cause damage to the host and trigger a waterfall inflammatory response. It has been reported that the activation of NLRP3 inflammasome is involved in the pathogenesis of pulmonary infections caused by *A. baumannii* (16). ROS have been shown to be important activation mediators of NLRP3 inflammasome (17), and the outer membrane protein 34 of *A. baumannii* stimulates ROS generation, leading to mitochondria damage and activation of the NLRP3 inflammasome in RAW264.7 cells, which exerts a key function in *A. baumannii*-induced pneumonia (18). Therefore, inhibiting the activation of NLRP3 and ROS is important for blocking *A. baumannii* infections (18). Our study found that *A. baumannii* prompted the activation of the NLRP3 inflammasome and the release of intracellular ROS through downregulating SIRT1 levels. This process can be inhibited and even reversed by L-serine. Additionally, L-serine inhibits the levels of inflammatory factors such as IL-18 and IL-1β that are stimulated by *A. baumannii*, further supporting the regulatory role of L-serine in *A. baumannii*-induced infections. On the other hand, L-serine improves mitochondria quality and repairs cell damage by repairing MMP and preventing excessive release of mtROS. L-Serine helps to reduce the levels of intracellular oxidative stress. Therefore, the stimulation of SIRT1 helps maintain the function of Beas 2B cells, which may explain why L-serine decreases the virulence of *A. baumannii* to Beas 2B cells in our study.

*In vivo*, the *G. mellonella* model was used to evaluate the virulence of *A. baumannii*. L-Serine markedly reduced the fatality rate of *A. baumannii* to *G. mellonella*. Although the effect of L-serine on the virulence of the remaining strains was not statistically significant, it significantly inhibited the corruption speed and degree of *G. mellonella*. Besides, L-serine decreased the virulence gene levels of *A. baumannii*. A study showed that *Omp*33-knockdown *A. baumannii* strains owned a low ability of adhesion and invasion to lung epithelial cells and expressed lower cytotoxicity (19). Sato et al. found that the virulence of *A. baumannii* varies with the levels of outer membrane proteins (OmpA, Omp33-36, and CarO) (20). It has been reported that outer membrane proteins (OMPs) of Gram-negative bacteria participate in regulating cell infection, biofilm formation, and antibiotic resistance (21). These studies indicate that OmpA and Omp33-36 are key virulence factors and are highly related to the invasion of *A. baumannii* strains. In our study, we observed that L-serine significantly decreased the *OmpA* and *Omp*33-36 levels in all high-virulent and most low-virulent strains. Therefore, L-serine decreases the chance of *A. baumannii* invading epithelial cells, thus reducing further damage to Beas 2B cells by downregulating these gene levels. *CarO* may also play an important role in the virulence of *A. baumannii*. Labrador-Herrera et al. observed that in a mouse model of peritoneal sepsis, the invasion of *A. baumannii* strains lacking *CarO* to human lung epithelial cells and the speed of invasion to important organs were low (1). In our study, *carO* levels were also downregulated significantly by L-serine. All these results prove that L-serine has the potential to reduce the virulence of *A. baumannii*. However, further studies with gene-mutant strains are necessary to elucidate the underlying molecular mechanisms by which these virulence genes contribute to the pathogenic processes.

**Fig 11:**
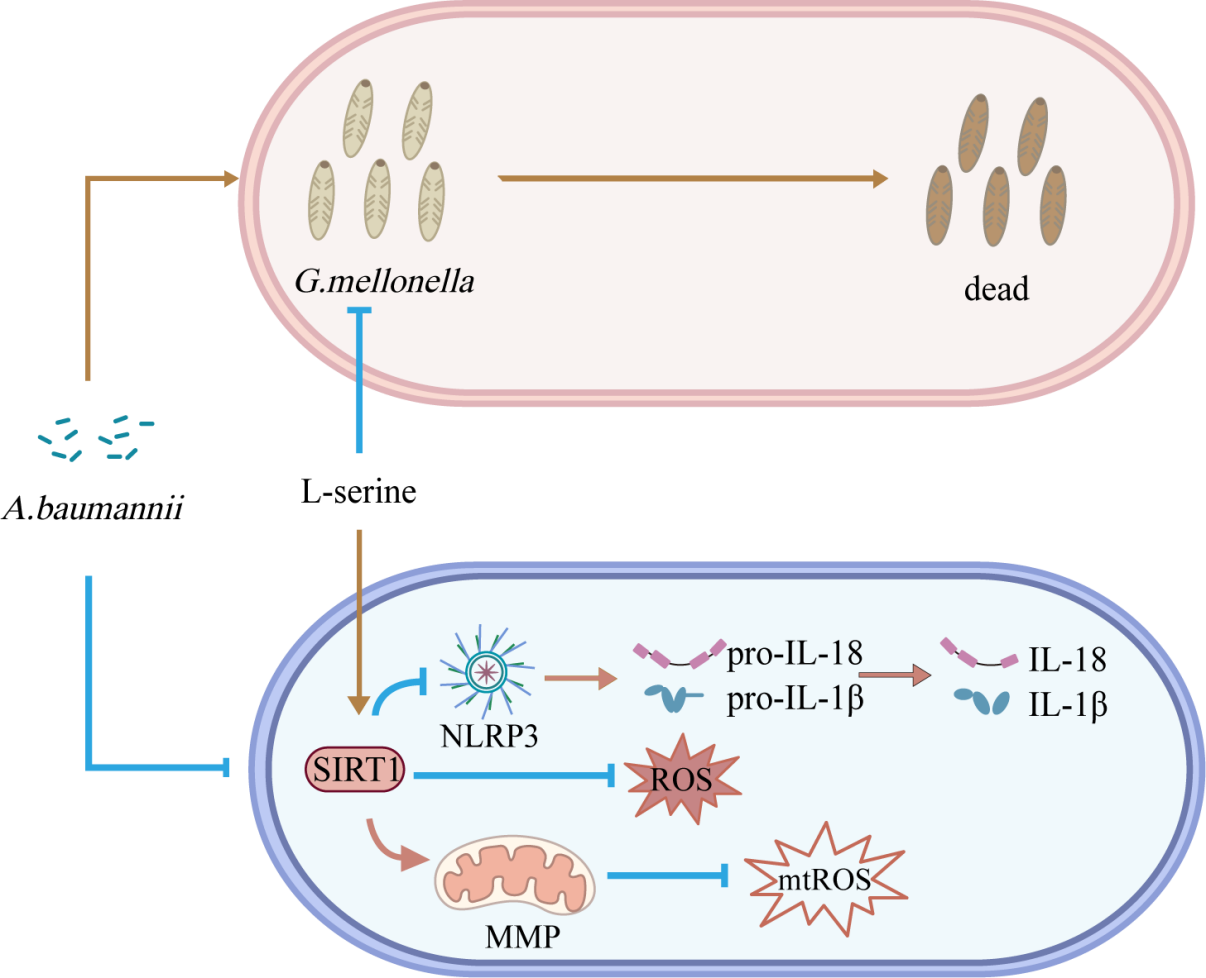
L-Serine reduces the virulence of *A. baumannii. A. baumannii* inhibits the expression of SIRT1, activates the NLRP3 inflammasome, and promotes the release of IL-18 and IL-1β in Beas 2B cells. Additionally, *A. baumannii* increases the generation of ROS, causes damage to mitochondria, and promotes the release of mtROS. *A. baumannii* also induces high mortality rates in *G. mellonella*. Treatment with L-serine reverses all of these processes and decreases the mortality rates of *G. mellonella* caused by *A. baumannii*.

Altogether, the development of treatments targeting virulence may provide a substitute for the increasingly failed traditional antibacterial treatment (22). Our current study reveals the inhibitory mechanism of L-serine on the infections caused by *A. baumannii* in Beas 2B cells and evaluates its effect on the virulence of *A. baumannii* against *G. mellonella* (Fig. 11). L-Serine owns the ability to reduce the virulence of *A. baumannii* and is expected to be a new strategy for the treatment or adjunctive treatment of *A. baumannii* infections.

## MATERIALS AND METHODS

### Bacterial strains, cell lines, and culture conditions

*A. baumannii* strains were collected from patients at the Third Affiliated Hospital of Sun Yat-sen University, Guangzhou, China, from May 2021 to August 2022. The ATCC 19606 strain was obtained from laboratory storage, and they were all stored at −80°. The strains were cultured in fresh Luria-Bertani (LB) media at 37°C. The overnight bacterial culture was collected, washed with saline, and suspended in M9 medium for subsequent experiments. The high-virulent *A. baumannii* strain AB9 was identified by cell infection in our study.

Human alveolar epithelial cells (Beas 2B) were obtained from laboratory storage. They were cultured in RPMI (GIBCO) supplemented with 10% fetal bovine serum (FBS) and 1% penicillin and streptomycin. The cells were cultured in an atmosphere of 5% CO_2_ at 37°C and passaged three times a week to maintain optimal growth. To observe the infection process, the six-well plates were planted with Beas 2B cells (about 1 × 10^6^ each well); 10 µL of *A. baumannii* solution (1 × 10^7^) was added 24 hours later. To evaluate the effect of L-serine (Sigma Aldrich), the cells were treated with L-serine (4 mg/mL) for 24 hours. EX527 (MCE), a specific inhibitor of SIRT1, was dissolved in 1% DMSO (Beyotime) and added to the culture medium to reach a concentration of 10 µM. The blank control group, *A. baumannii* infection group, L-serine+*A. baumannii* group, and L-serine+EX527+*A. baumannii* group were set separately for the subsequent experiments.

### Bacterial sample preparation and GC-MS analysis

Both the sample bacterial preparation and GC-MS analysis were performed as described previously (23). Briefly, 10 high- and 10 low-virulent strains were cultured in the LB medium with 200 rpm at 37℃ until reaching an optical density at 600 nm (OD600) of 1.0. The aliquot of 10-mL cells was quenched with precooled methanol (Sigma Aldrich) and by ultrasonication. Ribitol (0.1 mg/mL, Sigma Aldrich) was added as an internal standard. The aliquot of the 500-µL supernatant was separated with 12,000 *g* at 4°C for 10 min and dried by a vacuum centrifugation dryer (Labconco). Methoximation–pyridine hydrochloride (Sigma Aldrich) was added to the dried fraction above and continuously shaken with 200 rpm at 30°C for 90 min. Eighty microliters of N-methyl-N-trimethylsilyltrifluoroacetamide (Sigma Aldrich) was added and incubated at 37°C for 30 min. The data were analyzed using an Agilent 7890A GC and an Agilent 5975C VL MSD detector (Agilent Technologies). The compounds were identified by Agilent Chrom Station software (Agilent Technologies) and the National Institute of Standards and Technology (NIST) library. Every sample was analyzed in duplicate.

### *Galleria mellonella* virulence assay

The *G. mellonella* virulence assay was carried out as described previously with a few modifications (24, 25). *G. mellonella* with 2 ± 0.05 cm in length and 250 ± 25 mg in weight and aged 5 weeks old were selected and stored in a 4°C refrigerator. They were placed in a sterile Petri dish with a diameter of 10 cm and covered with absorbent filter paper. These *G. mellonella* were fixed and injected with 10 µL bacterial suspension using a 25-µL micro-injector. They were put in a dark environment at 37°C and observed every 12 hours for a period of 96 hours. Worms were evaluated as death when they turned black and showed no response to external stimuli. Strains that caused ≥80% mortality and <80% mortality were judged as high- and low-virulent strains, respectively.

### Quantitative reverse transcription–PCR assay

RNA extraction (AG), reverse transcription (Takara), and DNA amplification (Vazyme) were carried out according to the steps provided by the kit. Briefly, the total RNA of *A. baumannii* and Beas 2B cells were first extracted. Next, the RNA was reverse-transcribed into cDNA using a pre-mixed quantitative reverse transcription kit in a 20-µL system. Finally, DNA amplification was carried out using a fluorescent PCR instrument. GAPDH and 16s rRNA were used as reference genes for Beas 2B cells and *A. baumannii*, respectively. The relative expression of target genes was expressed by 2^−ΔΔCT^. The primer sequence used is listed in Tables S1 and S2.

### Western blot

About 1 × 10^6^ Beas 2B cells were put in each well and lysed with 80 µL RIPA (KeyGen) buffer for 20 min at 4℃ and oscillated by vortex every 5 min. The bicinchoninic acid protein assay kit (Beyotime) was used to estimate the concentration of protein. Each sample was loaded with 30 µg of protein and subjected to sodium dodecyl sulfate–polyacrylamide gel electrophoresis. The protein was then transferred to a polyvinylidene fluoride (PVDF) membrane (Merck Millipore) and blocked in buffer at 37°C for 1 hour. Primary antibodies (SIRT1, 1:1,000, Abcam; NLRP3, 1:800, Novus; ASC, 1:1,000, Abcam; and caspase-1, 1:1,000, Abcam) were diluted and incubated overnight at 4°C. The membranes were then incubated with the secondary antibody at room temperature for an hour. Chemiluminescence imaging was used to visualize the membranes.

### Measurement of mitochondrial membrane potential

MMP was measured according to the mitochondrial membrane potential detection kit (JC-1, HUAYUN). JC-1 solution was added, mixed, and incubated in six-well plates together with 1 mL RPMI 1640 at 37°C for 20 min. The MMP was lost after the treatment of CCCP (10 µM) for 20 min. The CCCP group was used as the positive control in this study. A fluorescence microscope was used to observe fluorescent signals. Green fluorescence indicated a decrease in MMP, while red fluorescence indicated normal MMP.

### Determination of reactive oxygen species (ROS and mtROS)

Fluorescence probes were used to determine the levels of ROS and mtROS. For the detection of ROS, DCFH-DA (Solarbio) was diluted in DMSO and added to the culture medium at a final concentration of 10 µM. The cells were incubated at 37°C for 1 hour. For the detection of mtROS (BestBio), a preheated working solution containing probes was added to the plate and incubated in the dark for 30 min. Fluorescence images were observed under a fluorescent microscope and quantified by ImageJ.

### Immunofluorescence

Beas 2B cells were fixed with 4% paraformaldehyde (Servicebio) for 10 min and broke by 0.2% of Triton-X 100 (Solarbio) for 15 min. Bovine serum albumin (BSA) (5%, BioFroxx) was used to block the cells at a shaker for 1 hour. SIRT1 antibody (1:200, Abcam) was added and incubated at 4°C overnight. An anti-rabbit secondary antibody was added, and the nuclei were stained with DAPI (Beyotime). The fluorescence intensity of SIRT1 was observed under a fluorescent microscope.

### Enzyme-linked immunosorbent assay

The concentrations of IL-18 and IL-1β collected in cell supernatants of Beas 2B cells were quantified by ELISA kits (MEIMIAN) according to the instructions of the manufacturer. The absorbance at 450 nm was measured on a microplate reader.

### Cell counting kit assays

The CCK-8 assays were performed according to the instructions of the manufacturer. Cells were seeded in triplicate in a 96-well plate at a density of 5 × 10^3^ per well. The aliquot of 100 µL RPMI complete medium was added to each well and cultured for 24 hours. L-Serine was added to the treatment group (TG) for 24 hours [multiplicity of infection (MOI) = 10]. The control group (CG) was infected by *A. baumannii* only. A blank control group was set (BG). Three hours after the incubation with 10 µL CCK-8 (Beyotime), the OD at 450 nm was measured. Cell viability was calculated using the formula: (OD_TG_ − OD_BG_)/(OD_CG_ − OD_BG_) × 100%.

### Statistical analysis

The metabolomic data were normalized and analyzed by IBM SPSS Statistics 22, Simca-P+ 12.0, GraphPad Prism 8.3, and R software (R4.2.3). Pathways were enriched with MetaboAnalyst 5.0 (https://www.metaboanalyst.ca). The experiment data were analyzed by GraphPad Prism 8.3 and expressed as the mean ± SD. Differences among or between groups were compared using one-way ANOVA or *t*-test. Figures were combined by Adobe Illustrator CC 2018. The survival rates of *G. mellonella* were expressed by survival curves. All the experiments were repeated at least three times. Statistical significance was considered at *P* < 0.05.

## Data Availability

The metabolomic raw data were deposited to MetaboLights under identifier MTBLS8966.

## SUPPLEMENTAL MATERIAL

Supplemental material spectrum.03226-23-s0001.doc Tables S1 and S2; Fig. S1 and S2.

ASM does not own the copyrights to Supplemental Material that may be linked to, or accessed through, an article. The authors have granted ASM a non-exclusive, world-wide license to publish the Supplemental Material files. Please contact the corresponding author directly for reuse.
