# Supplementary material for: L-Serine enables reducing the virulence of Acinetobacter baumannii and modulating the SIRT1 pathway to eliminate the pathogen
Source: Microbiol Spectr. 2024 Jan 19;12(3):e03226-23. doi: 10.1128/spectrum.03226-23 (PMC10913490; doi:10.1128/spectrum.03226-23)
Supplement: Supplemental material — Tables S1 and S2; Fig. S1 and S2. [file spectrum.03226-23-s0001.doc]

**Supplementary material**

Table S1. The primer sequence of genes in *A. baumannii* strains

| Genes | Sequence ( 5’-3’ ) |
| --- | --- |
| 16S rRNA | F:CAAAACTACTGAGCTAGAGTACG  R:TAAGATCTCAAGGATCCCAACGGCT |
| OmpA | F:AACAAATCAAACATCAAAGACCAA  R:GGTATTCAGATAATTTTTCAGCAACTT |
| Caro | F:AGCTTTACTTGCTGCTGGTG  R:CGAGCGCCTACTGGAATTA |
| Omp33-36 | F:CAAGTGTTGCTAACCAATTCGCT  R:GTTTTCTTGACCGAATGCACC |

Table S2. The primer sequence of genes in Beas 2B cells

| Genes | Sequence ( 5’-3’ ) |
| --- | --- |
| GAPDH | F:GGTGAAGGTCGGAGTGAA  R:CAGAAGGGGCAGAGATGA |
| SIRT1 | F:ATTCTTGTGAAAGTGATGAGGATG  R:ATTGTTCGAGGATCTGTGCC |
| NLRP3 | F:CCATCGGCAAGACCAAGA  R:ACAGGCTCAGAATGCTCATC |
| ASC | F:AACCCAAGCAAGATGCGGAAG  R:TTAGGGCCTGGAGGAGCAAG |
| Caspase-1 | F:CAAGGGTGCTGAACAAGG  R:GGGCATAGCTGGGTTGTC |
| IL-18 | F:TTTTGCCAAGGAGTGCTAAAGA  R:AACCCTCTGCACCCAGTTTTC |
| IL-1β | F:CTTTGCCGATCCGCCGC  R:ATCACGCCCTGGTGCCTGG |


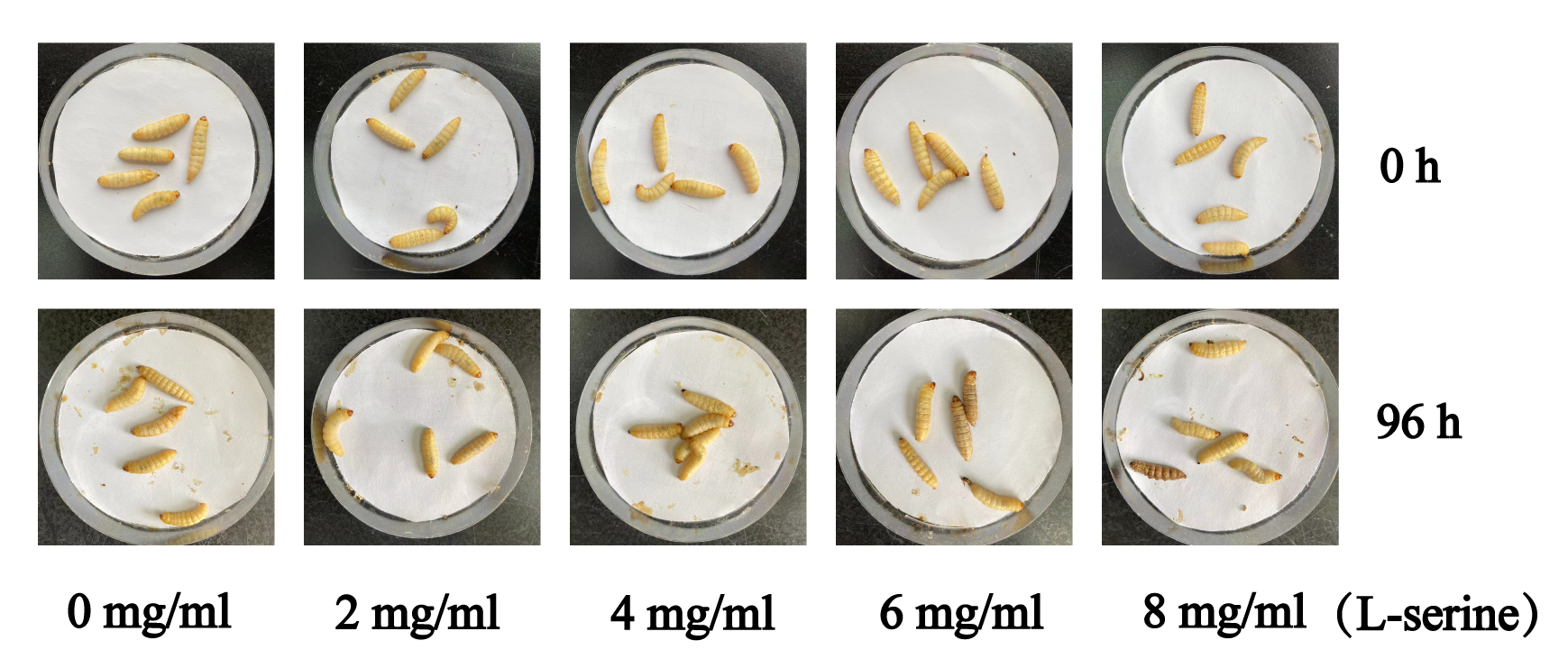


Fig.S1. The effect of L-serine on the activity of *G. mellonella*. Different concentrations of L-serine ( 2mg/ml, 4mg/ml, 6mg/ml, and 8mg/ml) were tested. After 96 hours, it was observed that 2-6 mg/ml of L-serine did not have any effect on the activity of *G. mellonella*. However, 8mg/ml of L-serine resulted in a reduction in the viability of *G. mellonella*. Therefore, 4mg/ml of L-serine was selected for further experiment.


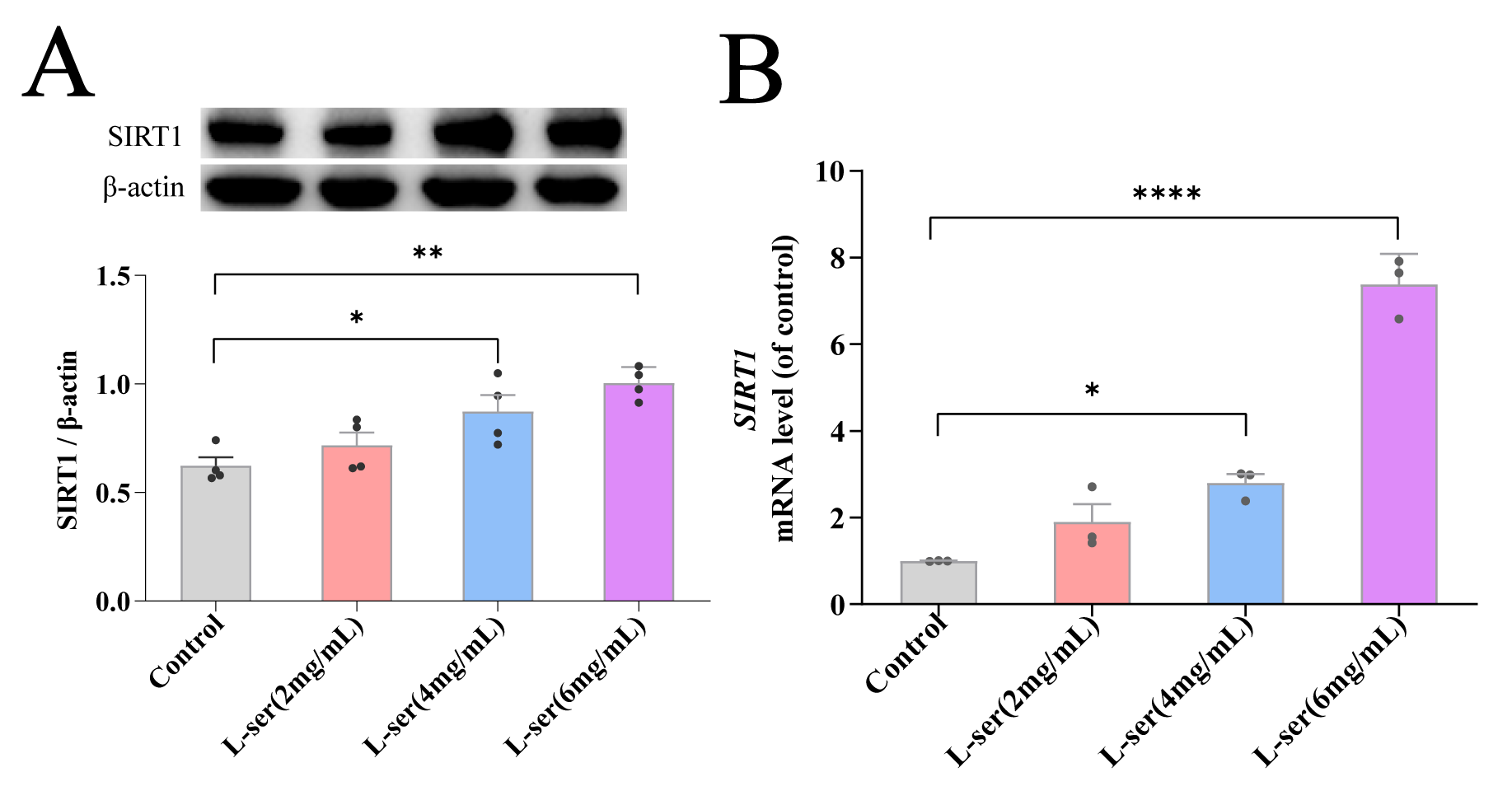


Fig.S2. L-serine increases the expression levels of SIRT1 in Beas 2B cells. (a) The effects of different concentrations of L-serine on SIRT1 at the protein level. (b) The effects of different concentrations of L-serine on SIRT1 at mRNA level. ^*^ P<0.05. ^**^P<0.01. ^***^P<0.001.
